# Supplementary figures and images for: Resilience of the Skin Microbiome in Atopic Dermatitis During Short-Term Topical Treatment
Source: Int J Mol Sci. 2025 Dec 4;26(23):11737. doi: 10.3390/ijms262311737 (PMC12692048; doi:10.3390/ijms262311737)

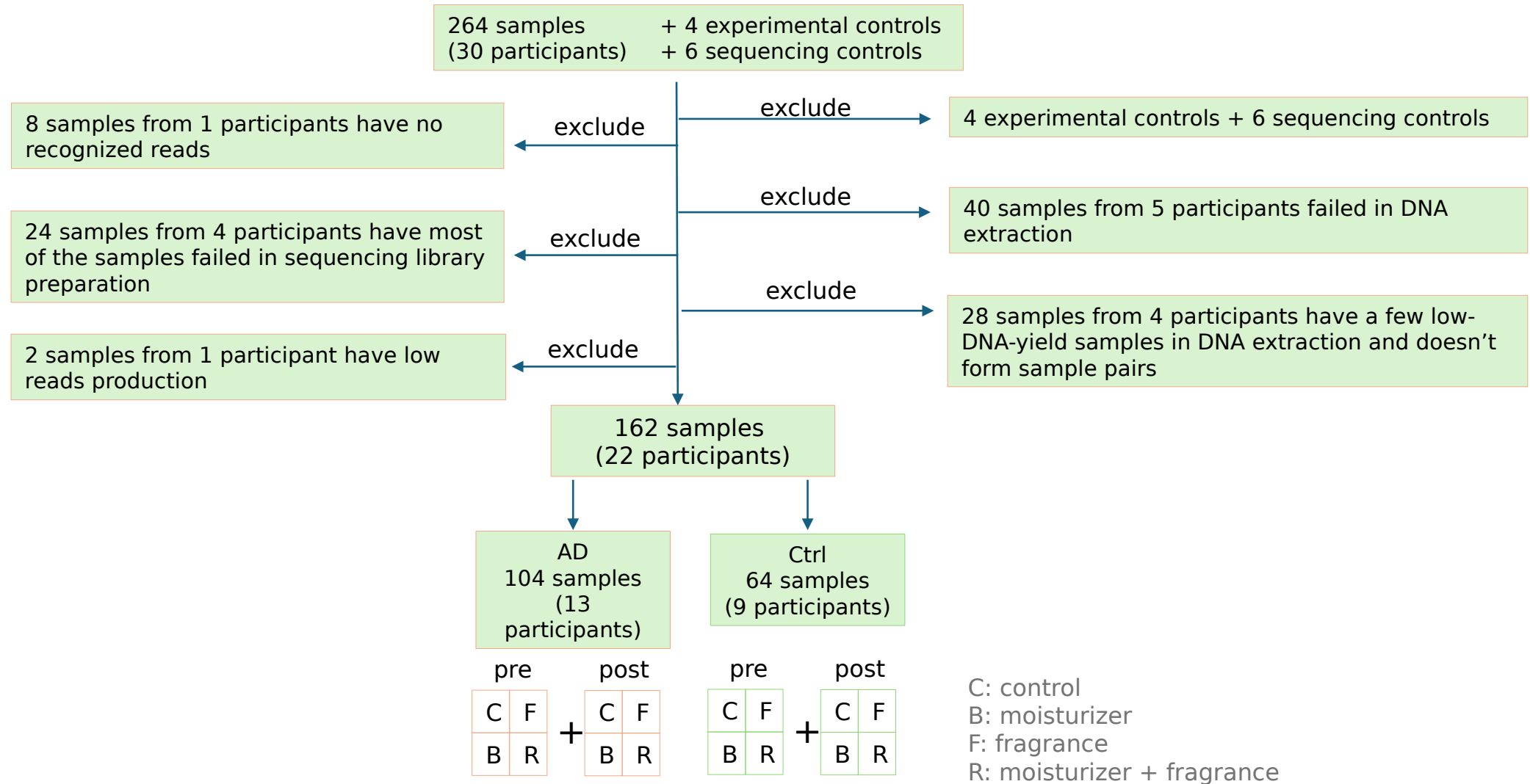

Supplement: Supplementary file 1 [file ijms-26-11737-s001.zip › sup.Fig1.pdf]

AD cohort

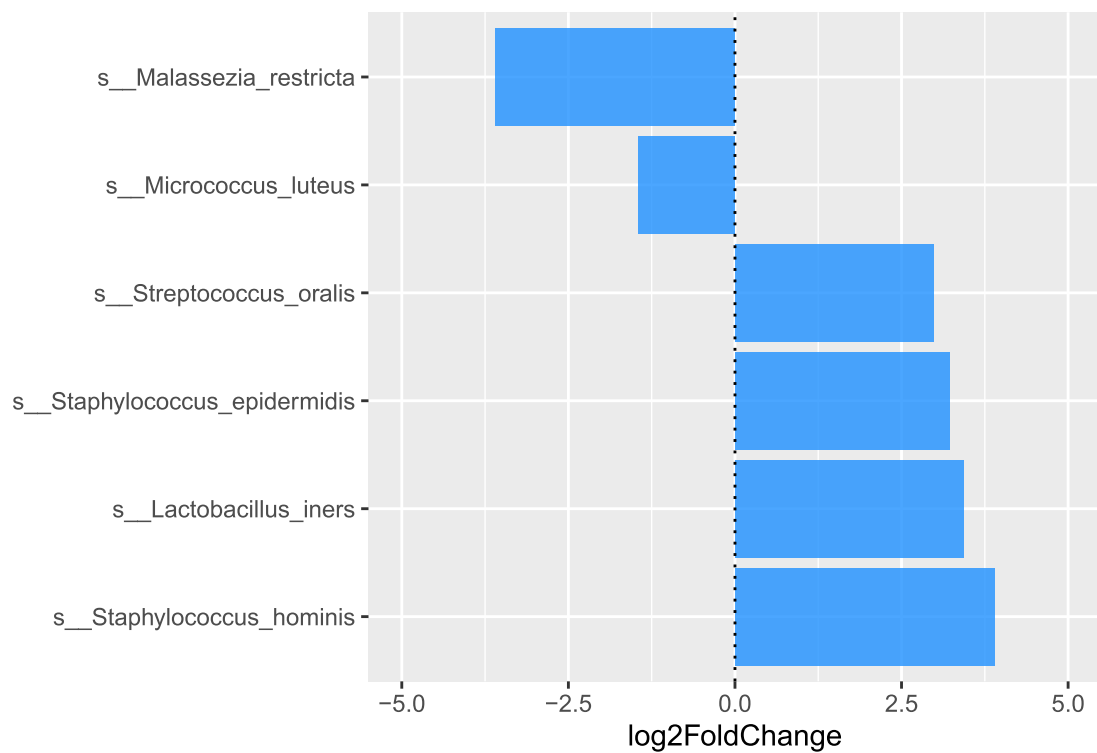

C vs M

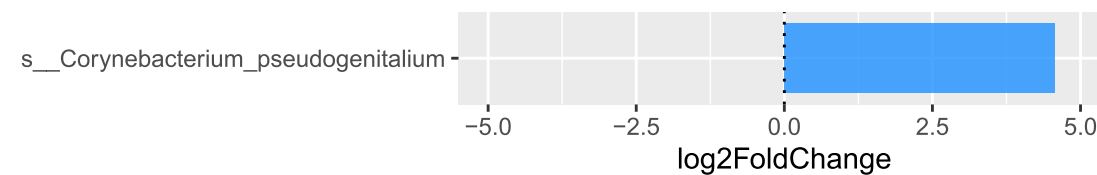

C vs F

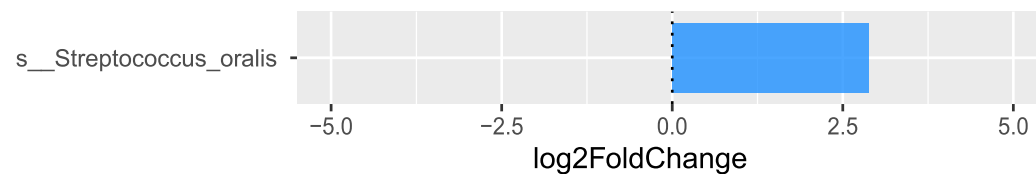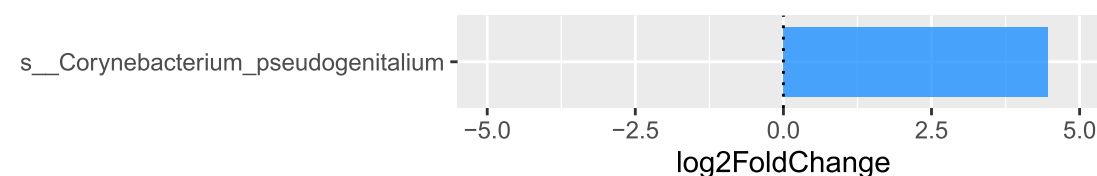

C vs MnF

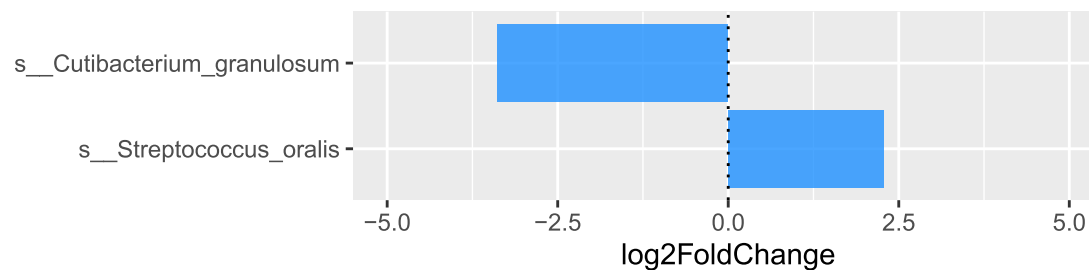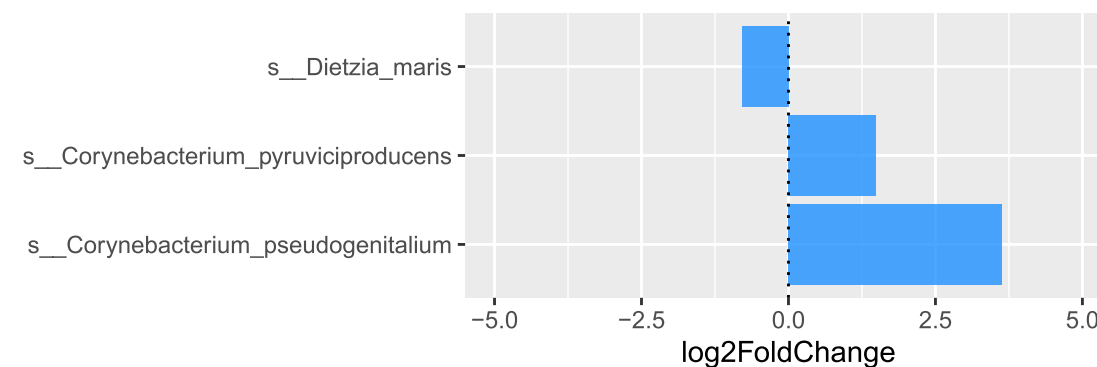

Supplement: Supplementary file 1 [file ijms-26-11737-s001.zip › supFig2.pdf]
